# Analysis of risk factors for intra-cystic hemorrhage in microwave ablation of partially cystic thyroid nodules

**DOI:** 10.3389/fendo.2023.1171669

**Published:** 2023-07-13

**Authors:** Yao Fu, Yuhui Huang, Yongtai Liu, Yu Song

**Affiliations:** ^1^ Department of Ultrasonography, the Second Hospital of Dalian Medical University, Dalian, China; ^2^ Department of Ultrasonography, Beijing Chao-Yang Hospital, Capital Medical University, Beijing, China; ^3^ Department of Acute Abdomen Surgery, the Second Affiliated Hospital of Dalian Medical University, Dalian, China

**Keywords:** ultrasound-guided microwave ablation, mixed cystic and solid thyroid nodule, contrast-enhanced ultrasound, intra-cystic bleeding, intracapsular hemorrhage

## Abstract

**Objective:**

The aim of this study is to identify risk factors of intra-cystic hemorrhage in microwave ablation of mixed solid and cystic microwave ablation s, and to design a preoperative nomogram to predict the risk value of intraoperative bleeding with the goal of individualizing the surgical approach toward different types of cystic and solid thyroid nodules.

**Methods:**

A total of 241 patients with cystic-solid thyroid nodules who underwent ultrasound-guided percutaneous microwave ablation were retrospectively divided into a bleeding group and a non-bleeding group to compare the diameter, cystic proportion, cystic fluid nature, color Doppler flow imaging, Contrast-enhanced ultrasound (CEUS) findings, and operative methods. Based on univariate and multivariate analysis, the important risk factors of nodular intracapsular hemorrhage in the ablation procedure were projected to a nomogram for predicting the possibility of intraoperative hemorrhage in the thyroid cystic solid nodules.

**Results:**

Intra-cystic hemorrhage was developed in 37 cases during the ablation of mixed thyroid nodules with a total incidence of 15% (37/241). Significant differences were found statistically between the two groups on the diameter of the lesions, CEUS findings, the cystic fluid ratio, and operative methods (*P* = 0.000, *P* = 0.001, *P* = 0.024, *P* = 0.002). The possibility of intraoperative nodular intracapsular hemorrhage was predicted by the model based on the risk factors with the accuracy of 81% and prediction consistency index (C-index) of 0.78.

**Conclusion:**

A new and efficient prediction model was developed based on the identified risk factors for intracapsular hemorrhage during microwave ablation of mixed thyroid nodules, which will aid in the development of targeted surgical planning for different types of cystic thyroid nodules, thus reducing the risk of hemorrhage during ablation.

## Introduction

A thyroid nodule has been discovered in more than 70% of the general population with a low incidence of malignant pathology (1, 2). Most patients undergo aggressive treatments for enlarged nodules (≥4 cm) that result in symptoms secondary to local mass effect (3). Thyroidectomy, through either open or endoscopic approaches, has long been the first-line choice as a definitive measure for this disease. Nevertheless, thyroidectomy not only carries various intra- and postoperative adverse risks (4), but also cannot eliminate the need the risk of re-operation in the case of recurrence (5, 6). As a result, various minimally invasive interventions, including ethanol ablation (7), laser ablation (8), microwave ablation (MWA) (9), and radiofrequency ablation (10) have emerged for managing large symptomatic benign thyroid nodules.

Microwave ablation has emerged as a preferred treatment methods, due to its many clinical advantages. The mechanism of treatment involves destruction of tumor via thermal stress, resulting in *in-situ* inactivation of the thyroid nodules. This results in less tissue carbonation, stronger coagulation, a larger destruction range, shorter ablation time, more uniform ablation, the limited cooling effect from blood perfusion, and more cost-effectiveness, all of which, thus have made MWA outperform traditional surgery in the treatment of benign solid or solid-cystic thyroid nodules (11). A recent study showed that the ultrasound-guided ablation achieved a one-year nodule reduction rate of 82.5–90.0%. Moreover, the application of mobile ablation and water isolation technology along with the empirical accumulation of operators have gradually lowered the complication rates related to the procedure, and have increased the safety and reliability of the operation (12–16).

Common procedure-related complications of thyroid nodule ablation are hoarseness, nodule rupture, and hemorrhage (17). Among these, intraoperative hemorrhage can lead to eventual hematoma formation, thereby prolonging the operation time and delaying the postoperative recovery (18). Since only a handful reports are available on the methods regarding the management of cystic-solid thyroid nodules and prevention of intraoperative intra-capsular hemorrhage (13, 19), this study aims to analyze the risk factors for intra-capsular hemorrhage during complicated cystic-solid nodule ablation, and to design a working prediction model to help better design targeted ablation plans for different types of cystic-solid nodules.

## Methods and materials

### Patient selection

**Figure 1:**
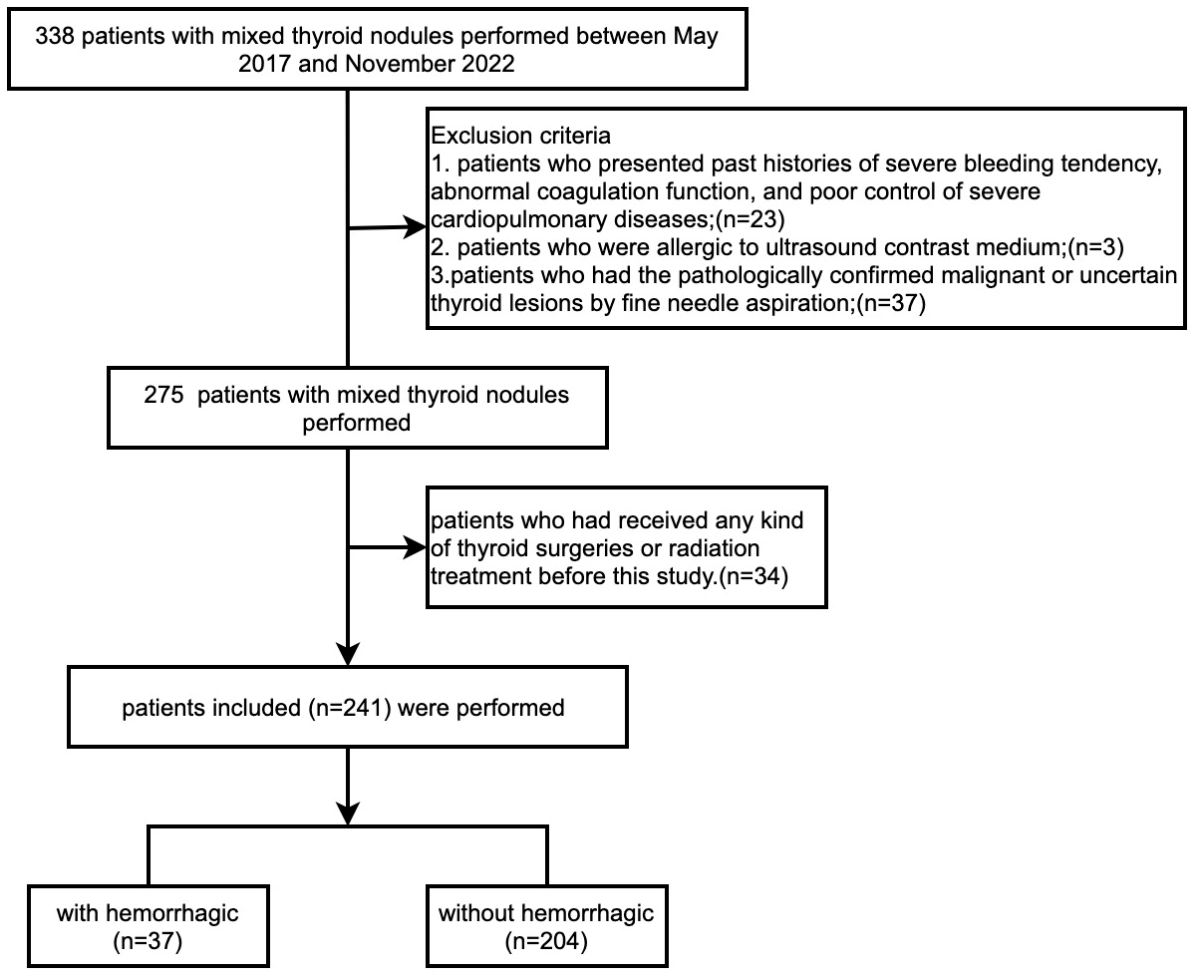
Flowchart shows study inclusion and exclusion criteria.

The present study retrospectively selected all the patients with the diagnosis of benign cystic-solid thyroid nodules who underwent ultrasound-guided microwave ablation treatment in our hospital from May 2017 to November 2022 (**Figure 1**). The inclusion criteria were listed as follows: (1) all patients undergoing both puncture cytology and liquid-based lamella cells for the preoperative pathological diagnosis; (2) thyroid nodule being greater than 2 cm at its longest diameter; (3) thyroid nodule being comprised of ≥20% cystic components. Moreover, patients who presented obvious symptoms as dyspnea or dysphagia or hoarseness or persistent anxiety about the possible malignant transformation were also candidates for the ablation procedure. The exclusion criteria included: (1) patients who presented any of the comorbidity of severe bleeding, coagulopathy, and poorly controlled severe cardiopulmonary diseases; (2) patients who were allergic to the ultrasound contrast medium; (3) patients who had the pathologically confirmed malignant or uncertain thyroid lesions by fine needle aspiration; (4) patients who had received any kind of thyroid surgeries or radiation treatment before this study. Moreover, the institutional review board of our hospital had reviewed and approved the study, and all participants had signed informed consent of procedures.

### Microwave ablation system and ultrasound guidance

All thyroid nodular lesions were ultrasonographically measured by using either MyLab™ Twice (Esaote, Italy) or Logic E9 (GE, USA) scanners equipped with LA523 and ML6-15 high-frequency linear array probes. Before the ablation, the microwave system ECO-100A1 (Yigao, China) was set at a frequency range in between 915MHz and 2450GHz for generating maximum temperatures of approximately 140°C. During the ablation, the probe was powered with 40 W to maintain the ablation temperatures above 100 °C, minimizing tissue vaporization and carbonization according to the manufacturer’s recommendation. Additionally, the contrast agent used for enhancing sonographic imaging was SonoVue (Bracco Imaging SpA, Milano, Italy) dry powder, which was further made into a microbubble suspension with infusion of 5 ml of normal saline before its use (**Supplementary Materials** for details).

### Pre-ablation evaluation

All the patients underwent thorough preoperative ultrasonographical studies to identify and analyze the nature, location, size, number, and the blood supply of the nodule, all of which helped to tailor the operating procedure for avoiding the injuries toward important blood nerves and vessels around the nodule. Specifically, the mechanical index was adjusted as 0.07, and both peripheral and internal enhancement patterns of the nodule were carefully assessed during contrast-enhanced ultrasonography.

### Operative procedure

**Figure 2:**
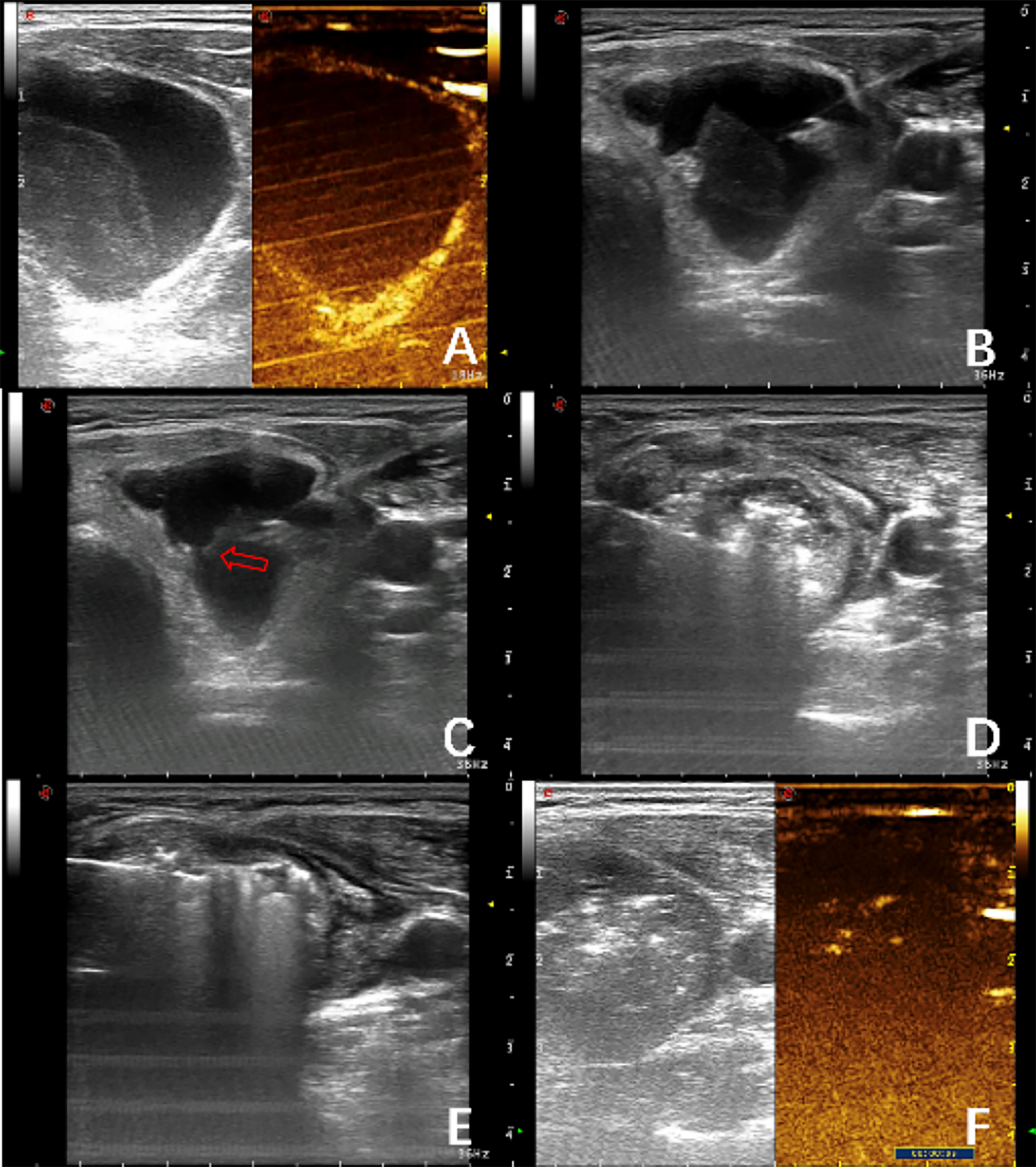
A 50-year-old woman presented with cystic solid nodules in the left lobe of the thyroid gland, about 4.1 cm × 3.7 cm × 2.8 cm in size. 70%, preoperative contrast-enhanced ultrasound showed abundant blood supply in the cyst wall and the internal solid part of the nodules and the contrast-enhanced image showed equal enhancement. **(A)** Aspiration of cyst fluid. **(B)** The nodules were bleeding in the cyst, and the bleeding spots on the cyst wall were squirted (arrows). The deflated cyst cavity was filled again, and the cavity was clouded **(C)**. Hematoma formed inside the nodules **(D)** Mobile layer-by-layer microwave ablation **(E)** was performed for solid components of nodules and hematoma. Immediate postoperative angiography, no contrast filling in and around the nodules **(F)**.

The ablation procedure was conducted in the hybrid operating room with the implementation of ultrasonographic equipment. The patients were placed in a dorsal decubitus position with the hyperextension of the neck in between the ultrasonographic device on the left side and the ablation instrument on the right side. After peripheral intravenous access was established, lines were connected for intraoperatively monitoring vital signs, and the surgical field was sterilized with iodine. The ablation procedure was initiated only under local anesthesia with the injection of lidocaine through the skin and into the deep fascial planes to the level of the anterior thyroid capsule. At this moment, the ultrasound scanning with a sterile probe was performed over the operating area to re-evaluate all critical structures (**Figure 2**), including the trachea and neck vessels in order to design a safe insertion path for the ablation needles so as to prevent vascular injuries from the puncture. If a thyroid nodule possesses cystic-solid nature with the cystic fluid as a jelly-like substance, the fluid can be aspirated after the injection of 0.9% normal saline to reduce the volume of the mixed lesion.

Subsequently, the microwave needle was forwarded to the targeted place to touch off real-time US-guided ablation on the lesion. Intraoperatively, patients are frequently asked to speak for monitoring any voice changes. When the nodule was small, the fixed ablation method was used. The extension of the ablation zone corresponded to the echogenic change around the antenna, and thus, if the hyperechoic zone failed to completely cover the nodule, the needle would be readjusted to achieve complete coverage of the target tissue by the ablation zone. After removing the ablation needle from the puncture site, compression was applied on the site for 2–3 minutes. In the case of multiple lesions, the ablations were performed on the unblocked nodules first, and then on the remaining nodules one by one. For nodules larger than 5 cm, in atypical locations, or located retrosternally, MWA was often performed in stages in order to avoid the development of complications. Following the ablation procedure, contrast-enhanced ultrasound can be performed again, and an additional round of ablation can be completed if the contrast is visualized to flow into the surgical bed. Afterward, all patients underwent local compression on the neck for 30 minutes with ice bag to relieve pain and reduce edema, and then were closely monitored for more than 2 hours before being discharged to home.

Intraoperative intra-cystic bleeding usually occurs from puncturing the thyroid, aspirating cystic fluid to deflate cyst cavity, and penetrating the nodule. Therefore, the puncture point should be first compressed for 3–5 minutes after the procedure to prevent bleeding. During the operation, If bleeding was not stopped after 2–3 rounds of repeated aspiration, the source of the bleeding could be ablated under ultrasound guidance to achieve hemostasis. For intracapsular hematomas that could not be aspirated, ablation will be performed on the solid component of the nodule.

### Image analysis

Adler grading method was used for visualizing the blood flow signals to and around the nodules with the doppler ultrasound, and in further evaluating intracystic hemorrhage, which commonly presented after aspirating or the ablation needle being inserted into the nodule, showing high or isoechoic punctate and mass signals in the nodule, and occasionally unveiling jetting signals as the bleeding site from the cyst wall.

### Statistical analysis

All statistical analyses were performed using SPSS 26.0 version. All the numerical data were expressed as the mean ± standard deviation. An independent sample t-test was used to compare the data between the two groups, and the chi-square test was applied to compare categorical data of two groups. The spearman correlation coefficient was used to analyze the correlation between contrast enhancement mode and bleeding rate, and the univariate analysis followed by multivariate logistics regression model were performed to establish a nomogram for predicting the risk factors of intraoperative bleeding by using R3.6.1 software along with the calculation of c-index. A *P*-value less or equal to 0.05 (*P* < 0.05) was considered statistically significant.

## Result

### General comparisons

The study collected a total of 241 patients with benign cystic-solid thyroid nodules undergoing ultrasound-guided microwave ablation in our hospital from May 2017 to November 2022. These patients had 49 males and 192 females with the age of 15–83 (50 ± 13) years old, and were further categorized into a bleeding group with intra-cystic hemorrhage (37, 15%), and a non-bleeding group (204, 85%). The further comparison did not find any significant difference in either gender, or cystic fluid properties, or CDFI grade between the two groups (*P* > 0.05), but exhibited that the length and proportion of cystic of nodules in the bleeding group were larger than those in the non-bleeding group, the operating methods of aspiration-ablation or ablation-aspiration-ablation were more common (*P* < 0.05), as shown in **Table 1**.

**Table 1:** Comparison of general data between the bleeding group and non-bleeding group.

Characteristics	bleeding group	non-bleeding	χ^2^/*t*	*P value*
	n=37	n=204		
**Gender, cases** **Age, years** **Nodules diameter** **Proportion of cystic fluid** 21%-50% 51%-70% 71%-99%	9/28 51 ± 10 3.77 ± 0.98 3 (3/37, 8%) 15 (15/37, 41%) 19 (19/37, 51%)	40/164 49 ± 13 3.08 ± 0.757 45 (45/204, 22%) 45 (45/204, 22%) 114 (114/204, 56%)	0.430 0.933 4.800 7.656	>0.05 >0.05 <0.01 <0.01
**CEUS** No enhancement Low enhancement Equal enhancement	5 (5/37, 14%) 5 (5/37, 14%) 27 (27/37, 73%)	66 (66/204, 32%) 56 (56/204, 27%) 82 (82/204, 40%)	13.870	<0.01
**Operation method** Ablation infusion-ablation ablation-infusion-ablation	3 (3/37, 8%) 30 (30/37, 81%) 4 (4/37,11%)	42 (42/204, 21%) 159 (159/204, 78%) 3 (3/204, 1%)	9.670	<0.05
**CDFI**			3.254	>0.05
Grade 0 Grade 1 Grade 2 Grade 3 **Fluid properties** Clarified yellow Stale bloody gelatinous None	3 (3/37,8%) 17 (17/37,46%) 11 (11/37,30%) 6 (6/37, 16%) 4 (4/37, 11%) 25 (25/37, 68%) 5 (5/37, 14%) 3 (3/37, 8%)	36 (36/204, 18%) 98 (98/204, 48%) 47 (47/204, 23%) 23 (23/204, 11%) 16 (16/204, 8%) 129 (129/204, 63%) 17 (17/204, 8%) 42 (42/204, 21%)	4.423	>0.05

(CDFI was classified by Adler classification method: Grade 0: no blood flow signal; Grade 1: sparse blood flow, punctate or short line of blood flow, its long diameter does not exceed 1/2 the diameter of the nodule; Grade 2: Usually 3-4 punctate blood flow or a long vessel penetrating the lesion, the length of the diameter is equal to or greater than 1/2 of the nodule; Level 3: enriched blood flow signal).

### Univariate analysis

The univariate analysis did not find significant differences in age, gender nodular cystic fluid properties, and CDFI between the bleeding group and the non-bleeding group (*P* > 0.05), but unveiled the differences in nodule diameter, contrast-enhanced ultrasound findings, the proportion of cyst fluid, and the operation method between the two groups (*P* < 0.05). The diameter of nodules in the bleeding group ranged from 2.0 to 6.3 cm (3.77 ± 0.98), which trended higher than that in the non-bleeding group (2.0–5.7cm), with an average of (3.08 ± 0.77) cm. In the bleeding group, the proportion of cystic fluid that occupied 51% to 70% of the lesion were found in 15 patients (15/37, 41%), and more than 70% were in 19 patients (19/37, 51%). 92% of intracapsular hemorrhage occurred in the mixed cystic and solid nodules with predominantly cystic content.

In the bleeding group, there were five cases (5/37, 14%) with no enhancement in the ultrasonographic images, five cases (5/37, 14%) with low enhancement, and 27 cases (27/37, 73%) with iso-enhancement. There was a positive correlation between the enhanced pattern of nodules and the incidence of intracystic hemorrhage (spearman correlation coefficient, r = 0.258) (*P* < 0.05).

### Multi-factor logistics regression analysis and nomogram risk prediction transformation

**Figure 3:**
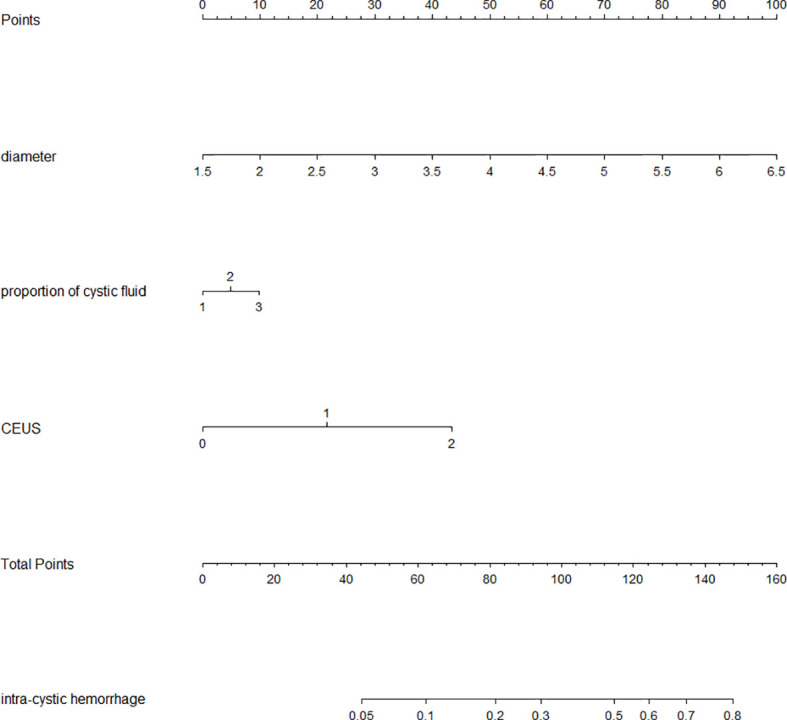
Nomogram of intraoperative intracapsular bleeding risk values. (Nodular cystic proportion 1: representing the capsule proportion of 21% to 50%; 2: representing the capsule proportion of 51% to 70%; 3: representing the capsule proportion of 71% to 99%; Contrast-enhanced ultrasound 0: no enhancement 1: low enhancement 2: equal enhancement).

**Figure 4:**
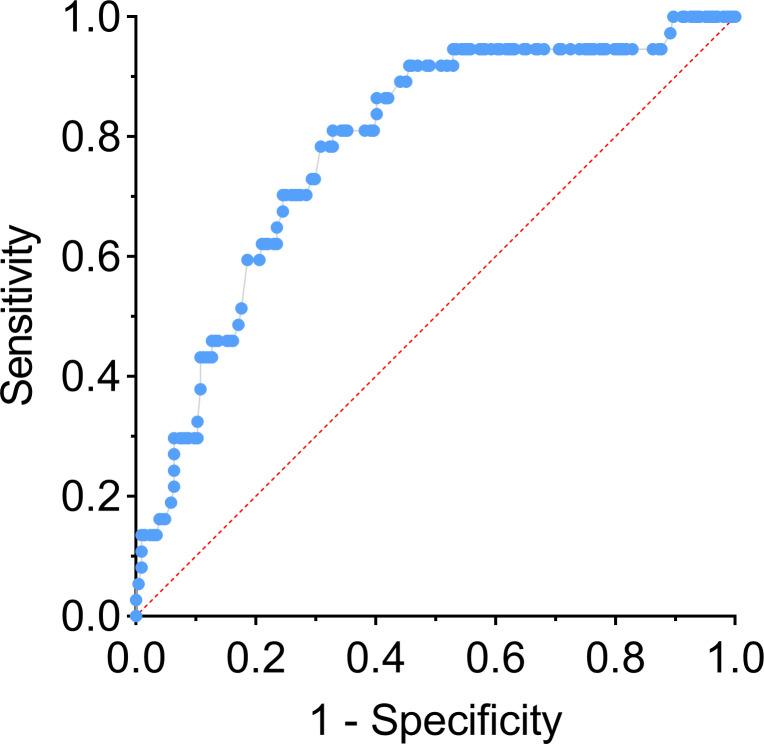
ROC curve of nomogram.

Multivariate Logistic regression analysis further identified the diameter of nodule and enhancement seen on ultrasound as independent influencing factors for bleeding (*P* < 0.05) (**Table 2**). Combined with previous literature (15) and clinical cases, the surgeons thought that the cystic proportion had strong clinical practical significance, and were compelled to include it in the multivariate statistical analysis. In the bleeding group, the nodules with a cystic proportion of 51%–70% accounted for 41% (15/37) of the total nodules, and the ones with cystic content of more than 70% accounted for 51% (19/37) of the total nodules. Notably,92% of intracapsular hemorrhage occurred in mixed cystic and solid nodules mainly composed of fluid. Furthermore, these three factors were ranked by the standard deviation along nomogram scales for the nomogram model as the longitudinal diameter of the lesion carrying highest risk potential (SD:0.238), the proportion of cystic fluid (SD: 0.714) being the second, and contrast-enhanced ultrasonography as the third (SD: 0.563). The total score was determined based on the individual scores calculated using the nomogram with the C-index value of 0.78 [95% confidence interval (CI) = 0.705–0.860], the accuracy of predicting intraoperative intracystic bleeding in nodules of 81%, and the area under curve of 0.782 [95% confidence interval (CI) = 0.705–0.859], altogether indicating good modeling for predicting subcapsular hemorrhage during MWA (**Figures 3**, **4**).

**Table 2:** Results of Logistic multivariate analysis.

Variable	Classified	*β*	SE	OR	95%CI	*P*
diameter		-0.8587	0.2377	0.424	0.266~0.675	0.0003
CEUS	0	0.5130	0.3731	4.376	1.453~13.181	0.1691
	1	0.4501	0.3676	4.109	1.394~12.115	0.2208
proportion of cystic fluid	1	0.9360	0.4433	2.786	0.687~11.291	0.0347
	2	-0.8474	0.3144	0.468	0.188~1.167	0.0070

## Discussion

The rate of thyroid nodules detected by high-frequency ultrasound is as high as 50%–76%, and the incidence of cystic solid nodules is the highest, accounting for 15%–53.8%. According to the American Thyroid Association guidelines, the risks of malignancy in purely cystic nodules, predominantly cystic nodules and predominantly solid nodules are about 1%, 3%, and 5%, respectively; additionally, spongiform morphology is highly specific for a benign nodule (20–22). Since most of cystic and mixed thyroid nodules pathologically possess benign nature, ultrasound-guided thermal ablation has been considered as the preferred choice when the enlargement of those nodules result in clinically significant symptoms (23, 24). A comparative study on thyroid thermal ablation versus thyroidectomy in 450 patients demonstrated that the thermal ablation was superior to conventional thyroidectomy in the categories of patient satisfaction, post-operative quality of life, and length of hospital stay (25). Furthermore, another study on MWA treatment of 474 benign thyroid nodules showed that the volume reduction rate of mixed cystic nodules (95% within 12 months) by the ablation was significantly higher than that of solid nodules, suggesting a curative effect was more definite in partially cystic thyroid nodules (15).

Importantly, MWA possesses more complexities in the treatment of thyroid cystic solid nodules than that of solid nodules, especially when intraoperative hemorrhagic occurs in the cystic lesion, which not only complicates the ablation, but also creates challenges in postoperative recovery. Thus, clinicians should pay extra attention to avoid the development of intracapsular hemorrhage during preoperative planning. This study analyzed the basic conditions of patients and the characteristics of nodules, which showed that larger cystic components of nodules contributed to a higher rate of intracystic bleeding during an ablation procedure. The diameter of nodules in the bleeding group of this study was consistent with that reported by Dong et al. (26), who reported the diameters of nodules ranged from 3.1 cm to 4.8 cm (average 3.9 cm). In addition, in the present study, 92% of intracapsular bleeding occurred in the mixed nodules predominantly with the cystic component, which was also in accordance with the study by Dong et al. (26), which found that all intraoperative bleeding nodules occurred in simple cysts or mixed nodule with more than 80% cystic proportion. Both our and Dong’s studies indicated that a higher cystic component in the mixed nodule resulted in higher risk in developing intracystic bleeding. Moreover, we found that iso-enhancing nodules accounted for 76% (19/25) of the total cases of hemorrhage, thereby suggesting that contrast-enhanced ultrasonography can be an effective method in preoperative preparation for predicting the bleeding risk. Following an extensive literature search for other similar studies, two articles by Kim et al. (17) and Agyekum et al. (18) were found to report the intraoperative complications related to cystic nodule ablation. However, none of these articles discussed intra-ablation hemorrhage in the cystic nodules. Our study has been the first one ever to investigate contributing factors to the development of hemorrhage and propose a prediction model for the ablation team to evaluate the bleeding risk of the cystic nodule in the thyroid in the process of ablation. 

A nomogram can visually represent the quantitative relationship between different predictor variables and target variables, and it can be used to predict the correlation extent of the incidence of an event and its potential risk factors. In this study, the top three contributory factors to the intra-lesion hemorrhage in the ablated mixed thyroid nodules were further ranked by their standard deviation along the nomogram scales in the nomogram model with the diameter of the nodules the cystic proportion of mixed nodules, and preoperative contrast-enhanced ultrasonography, which may more specifically direct the operators to take more strict and targeted operating procedures to prevent the intracystic bleeding from developing. Since our nomogram on these three risk factors carries the accuracy rate of 80.76%, the model can be efficiently translated into the daily practice on the MWA of mixed thyroid nodules.

Clinically, our study suggests that the ablation should be postponed in patients with a recent sudden increase in nodular volume or a history of hemorrhagic disease; technically, we emphasized that the needle tip should be placed far from the cyst wall, a small amount of fluid should be retained in the nodules with large cystic components to prevent the rupture of fragile vessels in the nodules after a sudden drop in pressure, and larger vessels should be avoided during ablation. For the nodules with rich blood flow, which are inevitably damaged during the procedure, we normally ligated the blood vessel first and drained the cyst fluid afterward. Intra-capsular hemorrhage that often occurred after the puncture and aspiration were completed or after the ablation needle entered the node presented with the expansion of the drained cyst cavity and could be managed with the compression on the puncture point for 3 to 5 minutes to prevent further extravasation, follow by aspiration of the remaining blood. If the hemorrhage continued after 2 to 3 aspiration attempts, additional ablations were performed with higher power at the suspected site of hemorrhage.

Based upon the findings in this study, the corresponding individualized ablation procedures are proposed for the nodules with different characteristics. First, for thyroid nodules with cystic component comprising 71%–90% of the total nodule volume, if the solid part or septa shows no or low enhancement by contrast-enhanced ultrasound, and the aspirated cyst fluid does not contain fresh blood, the operation can be performed with extracting cystic fluid first and then ablating the nodules thoroughly; if the above nodules are found to have jelly-like cystic material, the cystic material should be first diluted with the injection of a small amount of normal saline to alter the viscosity of the components in the cyst cavity, afterward, the mixed nodules can undergo MWA after the withdrawal of most of the cyst fluid; if a contrast-enhanced ultrasound showed solid components separated by equal enhancement with thick vessels on color Doppler imaging, the thick large vessels and the solid part of the nodules can be ablated first, then the cystic fluid in the nodule can be slowly aspirated, and finally the ablation part of the remaining nodule. For the thyroid nodules with 50%–70% cystic fluid, if the blood supply can be found in the solid part or septa by contrast-enhanced ultrasound, as much cystic fluid as possible can be aspirated prior to microwave ablation. If the solid part and septa contain a rich blood supply, the ablation needle cannot be inserted through the solid component; however, if the solid component can not be avoided, the ablation can be started once the needle tip reaches the nodal brim and continued through the solid portion of the nodules. Afterward, normal saline solution can be given to fill up the lesion, subsequently, the capsule of the lesion can finally be ablated. Third, for mixed nodules with cystic material of less than 50%, if the cyst fluid is gathered at the edge of the nodules, it can be aspirated first and then ablated the nodules, and if the cyst is dispersed (such as with spongiform nodules) or located in the central region of the nodules, it is difficult to extract fluid so such nodules can be directly ablated. Fourth, for the nodules with abundant blood supply and inevitable injury from puncture or ablation, vascular occlusion can be first performed followed by extraction of cyst fluid and then additional ablations.

The first limitation of this study is that the proportion of cystic components that could be aspirated was accurately evaluated by the volume of fluid, but for those mixed nodules, cystic fluid could not be completely aspirated or even could not be aspirated, the proportion of cystic content was only estimated based upon ultrasonographic measurement on the cystic component region, so the inconsistence in measurement should more or fewer influence results (27). Second, the long-term follow-up on the absorption of thyroid nodules was not done in this study, so the long-term outcome of MWA in the treatment of mixed thyroid nodules could not be fully established.

In conclusion, intra-cystic hemorrhage during microwave ablation of mixed thyroid nodules is not uncommon, so a feasible predictive model using identified risk factors can be used in preoperative evaluation for assessing the risk of intra-capsular hemorrhage, adding great value to clinical work in the form of improved prevention of operative complications.

## Data availability statement

The raw data supporting the conclusions of this article will be made available by the authors, without undue reservation.

## Ethics statement

The studies involving human participants were reviewed and approved by the institutional review board of the Second Hospital of Dalian Medical University. The patients/participants provided their written informed consent to participate in this study.

## Author contributions

YF: Validation, Formal analysis, Data Curation, Writing-Original Draft. YH: Investigation, Resources. Data Curation. YL: Visualization, Writing - Review & Editing. YS: Conceptualization, Methodology, Supervision, Project administration. All authors contributed to the article and approved the submitted version.
