# Supplementary material for: Analysis of risk factors for intra-cystic hemorrhage in microwave ablation of partially cystic thyroid nodules
Source: Front Endocrinol (Lausanne). 2023 Jul 13;14:1171669. doi: 10.3389/fendo.2023.1171669 (PMC10374254; doi:10.3389/fendo.2023.1171669)
Supplement: Supplementary file 1 [file Presentation_1.pdf]

Supplementary material

Microwave ablation system and ultrasound guidance

All thyroid nodular lesions were measured with both MyLab™ Twice (Esaote, Italy) and Logic E9 (GE, USA) scanners equipped with LA523 and ML6-15 high-frequency linear array probes. The ablation was performed with the cold circulation microwave treatment apparatus (ECO-100A1) (Yigao, China), which contains a generator, a flexible cable, and an antenna, and can convert electromagnetic energy in the form of high-frequency waves ranging between 915MHz and 2450GHz to thermal energy through the friction from the oscillation of water molecules. The generated thermal energy then dissipates around the tip of the antenna, in a variable ablation area determined by the power and the time of application for inducing temperature-dependent coagulative necrosis of the target tissue. Cellular destruction is caused by the denaturing of intracellular proteins and the cell membrane. When the tissue registers temperatures of 60 to 100 ℃, coagulative necrosis begins. Ablation time depended on the size and number of nodules. According to the standardized protocol in our hospital and the manufacturer's recommendation, we used 40 W power to maintain the ablation temperatures above 100 ℃ for minimizing tissue vaporization and carbonization. Its application in the clinical process can fulfill the objective. Additionally, the contrast agent for enhancing sonographic imaging was SonoVue (Bracco Imaging SpA, Milano, Italy) dry powder, which was formulated into a microbubble suspension with infusion of 5 ml of normal saline before its use.

logistic univariate analysis

Variable	Classified	*β*	SE	OR	95%CI	*P*
diameter		0.89	0.2101	2.435	1.613~3.676	<.0001
CEUS	0	-0.5444	0.354	0.23	0.084~0.63	0.124
	1	-0.3803	0.3552	0.271	0.098~0.747	0.2844
proportion of cystic fluid	1	-0.8416	0.4179	0.4	0.113~1.418	0.044
	2	0.7674	0.2929	2	0.935~4.276	0.0088
